# “A one-stop shop”: Real-world use and app-users' experiences of a psychoeducational smartphone app for adults with ADHD

**DOI:** 10.1016/j.invent.2025.100807

**Published:** 2025-02-04

**Authors:** Christina Seery, Rachel Hannah Cochrane, Martha Mulcahy, Ken Kilbride, Margo Wrigley, Jessica Bramham

**Affiliations:** a UCD School of Psychology, University College Dublin, Belfield, Dublin 4, Ireland; b ADHD Ireland, Carmichael House, North Brunswick Street, Dublin 7, Ireland; c HSE National Clinical Programme for ADHD in Adults, Health Service Executive, Dublin 8, Ireland

**Keywords:** ADHD, Adult ADHD, Digital mental health intervention, Psychoeducation, Real-world use

## Abstract

**Objective:**

Smartphone apps have the potential to be accessible tools to provide psychoeducation for adults with Attention-Deficit/Hyperactivity Disorder (ADHD). The Adult ADHD App aims to provide psychoeducation about ADHD and supports available in Ireland. The present study aimed to investigate acceptability and user engagement of the Adult ADHD App by auditing real-world use data and gaining qualitative feedback from app users.

**Methods:**

App analytical data was audited from the Google Analytics platform. Fourteen app-users, nine of whom identified as women and five as men, with an age range of 26–65 (*M* = 40.29, *SD* = 11.14), participated in semi-structured interviews. Reflexive thematic analysis was employed to develop themes on app-users' experiences.

**Results:**

The audit of app analytics indicated that over a period of one year, 12,000 people actively used the app and 6400 users returned after their initial use. We developed four themes from the interviews: ‘An evidence-based tool for understanding adult ADHD’ highlighted that the Adult ADHD App provides trustworthy psychoeducation on ADHD. ‘Recommendations for ADHD-friendly adaptations’ identified modifications to improve usability of the app. ‘Meeting the needs of different stages in the ADHD journey’ showed that while the app significantly benefitted adults new to their ADHD, adults who were familiar with their ADHD sought an app that would help them in moments of challenge. ‘Perception of public services impacts app experience’ suggested that people's attitudes and experiences of the public health system in Ireland affected their views of app content.

**Conclusion:**

The Adult ADHD App appears to meet its aims of providing psychoeducation on adult ADHD. Elements can be modified to improve usability. Digital health tools created by public health organisations should consider how people's previous experiences with healthcare services can impact how they perceive the information in the tools.

## Highlights

• Psychoeducation via smartphone apps may reduce the information gap for adults with ADHD
• The Adult ADHD App, a psychoeducational tool, was launched in November 2022
• The app was downloaded over 18 k times, with 12 k active users, within a one-year period
• App users, especially adults new to their ADHD, gained from trustworthy information

## 1 Introduction

Attention-deficit/hyperactivity disorder is a form of neurodivergence characterised by prominent features of inattention, hyperactivity, and impulsivity (American Psychiatric Association, 2013). Psychoeducation is recommended as a first step in providing supports to adults with ADHD (Kooij et al., 2019). There is increased interest in and recognition of adult ADHD in online communities, which may lead to further pressures on mental health services and increased waiting lists for ADHD services (Hartnett and Cummings, 2023). Therefore, accessible psychoeducation could help to reduce frustration while adults with ADHD wait for services as it may increase their understanding of ADHD and appropriate interventions (Gore et al., 2023). Mental health apps can offer wide-reaching access to psychological support and are immediately downloadable (Goldberg et al., 2022), as well as offering the opportunity to address information gaps in ADHD (Price et al., 2019). As such, apps could be a valuable tool to provide psychoeducation to adults with ADHD.

Smartphone apps have demonstrated satisfactory usability and effectiveness in supporting the psychological well-being of adults with ADHD (Knouse et al., 2022; Jang et al., 2021; Selaskowski et al., 2022; Selaskowski et al., 2023). For example, Knouse et al. evaluated the feasibility and usability of a cognitive behavioural therapy app, Inflow (Knouse et al., 2022). Inflow provides CBT-based psychoeducation and supports users to implement ADHD-specific cognitive and behavioural skills, as well as tracking their progress and engaging with other uses. They found that participants who did not drop out reported that Inflow was user-friendly, helpful and would recommend it to others, as well as demonstrating reductions in self-reported ADHD symptoms and functional impairment. The average user started three modules and completed two, indicating some drop-off in app use (Knouse et al., 2022). Similarly, Jang et al. explored the feasibility of a mobile app with chatbot-delivered psychoeducation related to CBT, Todaki (Jang et al., 2021). They also observed reductions in ADHD symptoms, and usage past the first week was significantly reduced.

Selaskowski et al. have conducted several studies evaluating smartphone-assisted psychoeducation not within the remit of a CBT intervention (Selaskowski et al., 2022; Selaskowski et al., 2023). In their initial study, they compared intervention groups supported by their psychoeducational smartphone app (‘AwareMe ADHS’) to groups with traditional pen-and-paper brochures. The app featured eight modules with informational content, homework assignments, and quizzes. They observed a greater reduction in ADHD symptoms in the smartphone-assisted group compared to brochures, and no significant differences in quiz performances between the groups. They did not measure the acceptability, with the primary indicator of feasibility being attrition, therapy compliance, and homework compliance. They observed no significant differences in attrition and therapy compliance, but the smartphone-assisted group submitted significantly more homework assignments (Selaskowski et al., 2022). However, this study evaluated the app in the context of a facilitated intervention, which does not provide insight into how adults with ADHD might use the AwareMe ADHS app themselves beyond a guided intervention. In comparison, their more recent work compared self-guided use of the app to a psychoeducational chatbot (Selaskowski et al., 2023). While both groups demonstrated improvements in ADHD symptoms and psychoeducational knowledge, they appeared to be as effective as one another. They did not report data on app use or participants' views of the app.

One of the informational gaps people with ADHD have identified is not knowing what services are available (Price et al., 2019). This information is likely country and even region-specific, as service delivery for ADHD varies significantly across Europe (Europe, 2020). The Inflow app provided information about executive functioning (Knouse et al., 2022). The AwareMe ADHS app similarly covered self-organisation, mood regulation and impulsive behaviours, while also providing information on basic information about ADHD, personal resources, mindfulness, stress management, and relationships (Selaskowski et al., 2022). The chatbot Todaki covered three major areas: checking for attention deficit symptoms compared to mood disorders; overcoming attention deficit or behavioural interventions, medication, emotion regulation, and mindfulness training; and exploring attention deficit (Jang et al., 2021). While all apps provide a wealth of knowledge related to ADHD, it may be valuable for a region-specific app to provide insight into the supports and services available locally for ADHD, and what interventions are provided, to address informational gaps.

Additionally, despite evidence of effectiveness from pilot studies and randomised controlled trials, digital mental health interventions often do not get fully integrated into healthcare settings (Mohr et al., 2017). Low adherence and attrition rates may undermine the apps' effectiveness (Linardon and Fuller-Tyszkiewicz, 2020). To address issues with low adherence and attrition rates, it is valuable to understand adults with ADHD's experiences of smartphone apps. Like the evaluations of facilitated psychoeducational interventions (Pedersen et al., 2024), studies evaluating psychoeducational smartphone apps for ADHD have mostly relied on self-report scales to explore usability and attrition to indicate acceptability or feasibility of the app (Knouse et al., 2022; Jang et al., 2021; Selaskowski et al., 2022). Jang et al. included open-ended questions on experience, which highlighted that users experienced unnatural conversations with Todaki and non-intuitive interfaces (Jang et al., 2021). Further qualitative evaluations of apps for ADHD could help to understand how adults with ADHD experience the app's usability, and their recommended modifications to increase their engagement with the app over time.

Several qualitative studies have explored adults with ADHD's use and perceptions of digital mental health tools. Analysis of interviews has highlighted the therapeutic mechanisms associated with psychoeducational content in an online intervention (Flobak et al., 2021). Additionally, qualitative research has provided insight into the barriers and facilitators to engagement for adults with ADHD, such as the impact of ADHD symptoms, difficulties with navigation and pathologising language (Denyer et al., 2023; Kenter et al., 2022). Qualitative research also offers an important opportunity to highlight and centre adults with ADHD's perspectives on the digital mental health tool as they have been historically side-lined by neurotypical developers (Spiel et al., 2022).

In response to a lack of services for adults with ADHD, the public health system in Ireland, the Health Service Executive (HSE), developed a model of care to establish a National Clinical Programme for ADHD in Adults. This aims to provide tertiary care to adults with ADHD by delivering assessments and treatment via ADHD clinics in eleven catchment areas across Ireland (Raaj et al., 2023). Five clinics were in operation at the time of the present study. Given the long waiting lists to access support for ADHD in Ireland (Europe, 2020) and the negative impacts of waiting lists documented by previous research (Punton et al., 2022; Reichert and Jacobs, 2018), the Adult ADHD App was developed to provide an accessible resource on ADHD for adults to access while waiting for services. It was developed as a collaboration between the HSE, ADHD Ireland, a voluntary charity organisation, and the first and senior authors' host institution. The free-to-use app aims to provide psychoeducation about ADHD, as informed by adults with ADHD's priorities for digital psychoeducational tools (Seery et al., 2022). The Adult ADHD App was launched in November 2022 and is available to download in the App Store and Google Play Market.

Given the novel nature of the app, investigating its acceptability and user engagement can identify if the app is meeting its aim of providing psychoeducation on adult ADHD, and potential improvements to usability and experience. Despite being a core aspect of digital mental health interventions, acceptability is a complex and nuanced concept that can be challenging to measure, and no consensus on its definition (Perski and Short, 2021). However, understanding aspects of acceptability is incredibly valuable, as it can predict and explain user engagement, intervention effectiveness, and implementation (Perski and Short, 2021). Sekhon and colleagues propose a framework of acceptability comprising prospective acceptability, concurrent acceptability, and retrospective acceptability. They suggest that there are seven constructs which can be relevant to each stage of acceptability: affective attitude, burden, ethicality, intervention coherence, opportunity costs, perceived effectiveness, and self-efficacy (Sekhon et al., 2017). In the context of smartphone apps for adult ADHD, these constructs might be evaluated using user engagement data, and qualitative data to provide insight in individuals' experiences of acceptability. The acceptability of intervention components and design elements will change over time and will be influenced by social and cultural contexts (Perski and Short, 2021). As such, it is not necessarily possible to ‘achieve’ acceptability, as the conventions will likely change. Instead, research can be used to gain insight into acceptability, such as what elements users perceived as effective and diverging opinions on app features and improve the suitability of an intervention.

Understanding the app's real use over time can provide insight into the behavioural components of user engagement, such as frequency, amount, depth, and duration of use (Perski and Short, 2021). Real-world use can also help understand acceptability, as the number of downloads versus active uses could represent prospective acceptability, while initial use levels might suggest concurrent acceptability. User retention and continued engagement can also suggest the app's sustained user acceptance (Perski and Short, 2021). Qualitative research can then further contextualise real-world use and provide insight into acceptability.

The present study aimed to explore the Adult ADHD App's acceptability and user engagement. App analytical data were audited to understand uptake of the app the year after its launch. Qualitative interviews were employed to explore app-users' overall experience, the factors that influenced their app use and their experience of app features and psychoeducational content.

## 2 Methods

This study audited app analytics to indicate the use of the Adult ADHD App over a one-year period since its launch and qualitatively explored app users' experiences. Ethical approval was granted by the first and senior authors' host institution (HS-21-23-Seery-Bramham). All participants provided informed consent.

### 2.1 The adult ADHD app

The Adult ADHD App is a psychoeducational resource for adults with ADHD. The app was developed to address the limited access to psychoeducation and gaps in information provision in the area of ADHD (Price et al., 2019; Matheson et al., 2013). There are significant delays in accessing ADHD-specific services, with waiting times of potentially 5–10 years for newly referred adults in the United Kingdom (Smith et al., 2024) and over four years in some services in Ireland (Pepper, 2024). As such, there are delays in accessing evidence-based, trustworthy psychoeducation about ADHD from clinicians and it is necessary to provide a stopgap by offering accessible psychoeducation. Apps are wide-reaching due to not being geographically limited like an in-person or service-specific psychoeducational group might be, and can be downloaded immediately (Goldberg et al., 2022). As such, the Adult ADHD App was developed to be a preliminary resource for learning about ADHD while an individual is waiting to access specialist services.

The app aimed to address a wide variety of information needs related to ADHD. Based on our findings from a Delphi-consensus study of what adults with ADHD wanted to know (Seery et al., 2022), we understood there to be broadly three areas of information needs: an introduction to ADHD or what it is; living with ADHD and supporting oneself; and understanding the Irish public health services for ADHD, including the referral process, available clinics, and interventions offered within the services. We hoped the app would serve as a helpful introduction to having ADHD as an adult in Ireland for individuals recently self-identified or diagnosed, to reduce the burden on individuals to scour the internet and evaluate whether the information they found was reliable. The app is advertised as an informational tool that provides psychoeducation on self-care and signposting for adult ADHD in Ireland. The app was part of a larger project to create accessible psychological supports for adults with ADHD, in which a novel online, facilitated intervention was developed and evaluated for individuals who required more input than the app (Seery et al., 2023; Seery et al., 2024). Therefore, while we were interested in user engagement in terms of length of time spent on the app, the primary indicator of success was if participants felt the app was beneficial in learning about ADHD.

A human-centred design approach was adopted (Sucala et al., 2020). The first phase consisted of identifying the priorities for psychoeducation and how the app should be presented (e.g., graphics and text) with experts by experience (Seery et al., 2022). Following this, the content was written and reviewed by psychologists working in adult ADHD. Stakeholders working in ADHD (e.g., multidisciplinary clinicians working in the public ADHD services, general practitioners and ADHD coaches working privately, and board members of ADHD Ireland) were then engaged to provide feedback on the app and its content, to ensure clinical accuracy. Following adaptations based on stakeholder feedback, members of ADHD Ireland were invited to provide feedback on a prototype of the app. Forty-nine individuals with ADHD completed an open-ended survey on their experience of the app. Participants of the end-user consultations were 75 % women, 19 % men, and 6 % non-binary people; 43 % aged 36–45, 30 % aged 26–35, 19 % aged 46–55, 6 % aged over 65, and 2 % aged 56–65; and 81 % formally diagnosed by a clinician and 19 % self-identified as having ADHD. Feedback was provided on the positive elements of the app, as well as suggestions for addressing the SMART-goals planner, increasing the suitability of the app for ADHD, more neuro-affirmative language, improving the user interface and accessibility, and clarifying how the app should be used. The feedback was incorporated and changes made accordingly (e.g., the mindful breaks were and infographics were added, as well as language adapted to be more neuro-affirmative throughout the app). The app was then launched in November 2022.

In the context of widespread misinformation about ADHD on TikTok (Verma, 2024; Pineda et al., 2023; Yeung et al., 2022), the app aimed to provide evidence-based psychoeducation, meaning all information in the app about ADHD, living with ADHD, and self-help tools drew on scientific sources, with references available if users wished to know more. For example, the section on ‘Exercise’ draws on research studies that show aerobic exercise boosts attention in people with ADHD (Mehren et al., 2019). The clinical content on the National Clinical Programme for ADHD in Adults in Ireland was based on the HSE's Model of Care and reviewed by several clinicians working within the ADHD services to ensure the accuracy and credibility of information.

The app is divided into five sections: about ADHD-related traits, self-help techniques, living with ADHD, seeking help, and services for adult ADHD in Ireland. In total, there are 37 static pages on the app. All pages are written materials, bar the mindful breaks and SMART goals-based planner. The mindful breaks are brief mindfulness audio practices tailored to ADHD (all under three minutes), with practices for getting started or overcoming procrastination, a self-compassion break, increasing focus, responding to difficult thoughts and emotions, and a three-minute breathing space. The pages about ADHD, self-help techniques, and living with ADHD are arranged into two parts: what is it and what can I do about it. This is to provide an understanding of the concept and how it's related to or why it is beneficial for adults with ADHD, and then specific techniques and tools that are helpful. All written content is formatted as bullet points, as advised by adults with ADHD (Seery et al., 2022). The pages for about ADHD and self-help techniques feature colourful infographics to make content more digestible.

**Fig. 1:**
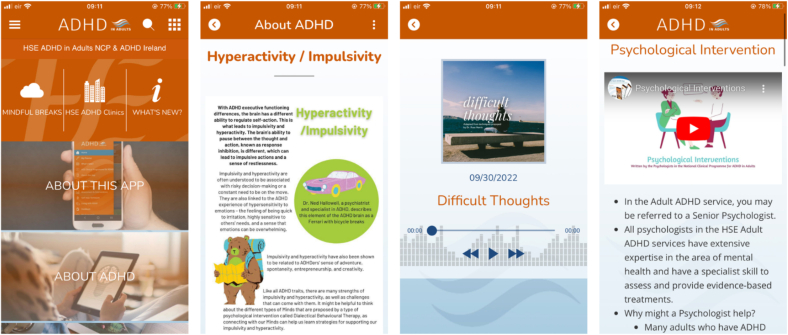
Example screenshots from the Adult ADHD App.

The app was arranged intentionally to follow the process of understanding and learning about ADHD, in an effort to meet the diverse needs of adults with ADHD. The first section ‘About ADHD’ is to introduce ADHD traits for individuals querying if they have ADHD or wanting to learn more to support a loved one. For those who resonate with the traits or are recently diagnosed, they can find information on tools to support themselves in ‘Self-Help’ techniques. Adults who are familiar with their ADHD and self-help tools but want more guidance can visit ‘Living with ADHD’, which features content on mental health (e.g., low self-esteem, co-occurring mental health problems, emotion regulation, addiction and sleep), relationships, accommodations at work and in education, and legal rights. Individuals who would like to access Ireland's public health services for ADHD can read about the process in ‘Seeking Help’ (which includes information on accessing a general practitioner in Ireland) and the clinical teams in ‘Interventions’. Information about which clinical teams are operational is included on the page ‘HSE ADHD Clinics’. Fig. 1 provides example screenshots from the app.

### 2.2 Procedure and participants

Participants were recruited via ADHD Ireland's mailing list and social media, in addition to a pop-up advertisement on the Adult ADHD App. Advertisements invited app-users to share their experiences of using the app. People interested in participating emailed the first author, who provided them with the information sheet, confirmed scheduling for the interview, and sought informed consent.

Semi-structured interviews conducted via Zoom were used to collect data (Zoom Video Communications, Inc. Zoom cloud meetings, 2023). Interviews were conducted by two research assistants who graduated from their undergraduate degrees in Psychology who were not involved in developing the Adult ADHD App. The interview questions were adapted from Arnold et al.'s semi-structured interview guide, as they conducted a similar study evaluating a web-based psychosocial intervention for people experiencing psychosis (Arnold et al., 2020). The interview schedule is available in the online supplementary material. Participants were fourteen adults with self-reported ADHD who had used the Adult ADHD App. Twelve participants reported a formal diagnosis of ADHD, and two self-identified as having ADHD and were undergoing the assessment process. Only four participants reported the time since diagnosis (*M* = 3.25, range: less than one year to eight years). Nine participants identified as women, and five identified as men, with an age range of 26–65 (*M* = 40.29, *SD* = 11.14). Interview length varied from 12 to 56 min (average length = 29.41).

Ingenium hosts the Adult ADHD App and uses the Google Analytics platform to collect, store, and analyse app data. The first author, CS, was given access to the Google Analytics. When logging into the app initially, users can consent to their app traffic data being collected. App data on user acquisition, engagement and retention between November 2022 to October 2023 was extracted from the host of the app's Google Analytics account.

### 2.3 Data analysis

App-use data was explored descriptively. Reflexive thematic analysis was used to inductively analyse the data collected from interviews (Braun and Clarke, 2006; Braun and Clarke, 2019). As analysis was influenced by research questions, this added a deductive element to analysis (Byrne, 2022; Braun and Clarke, 2012). We adopted a critical realist ontological approach and considered how participants' contexts and systematic structures may influence their interpretations of reality (Houston, 2001; Pilgrim, 2018). CS transcribed the interviews to familiarise herself with the data. Data were then coded by hand, using a semantic and latent approach (Byrne, 2022). All interviews were coded twice. Codes were reviewed and discussed with the senior author, JB, prior to being clustered together into initial themes. Themes and their names were then refined and developed by CS and JB and reviewed by the research team. Throughout the process of development and evaluation, CS reflexively journaled (Annink, 2017) and maintained an audit trail. She also discussed thoughts and assumptions with the senior author, the research team, and peers.

## 3 Results

### 3.1 Audit of app analytical data

**Fig. 2:**
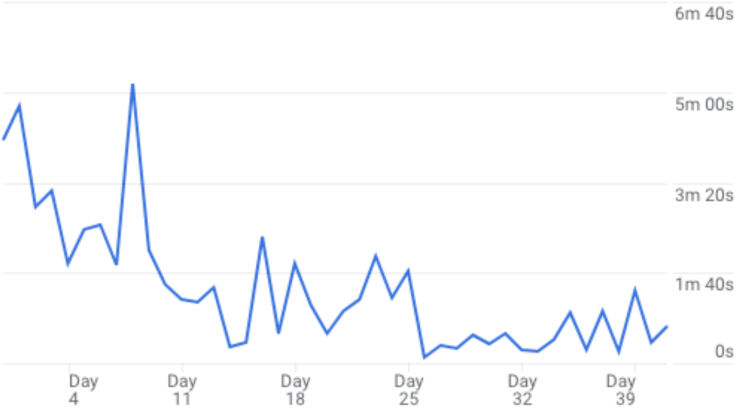
Visualisation of average user engagement across the initial forty days after download.

The Adult ADHD App was downloaded over 18,000 times between November 2022 and October 2023. Of these downloads, approximately 12,000 people actively used the app and around 6400 returned to the app. Users spent an average of 4 min and 55 s on their first use of the app. The average length of time users engaged with the app was 8 min and 58 s and the number of engaged sessions was typically 2.4 per user. Engaged sessions are defined as a session lasting longer than 10 s, with key events, and at least two pageviews (Google Analytics Help, n.d.), indicating active use. In total, there were 758 k user actions, such as clicking links in pages, navigating through the app, or engaging with mindful practices. Fig. 2 shows average user engagement across the initial forty days after downloading the app. As demonstrated by the visualisation, engagement peaked in the first two weeks after download.

### 3.2 Reflexive thematic analysis of app-users' experiences

**Fig. 3:**
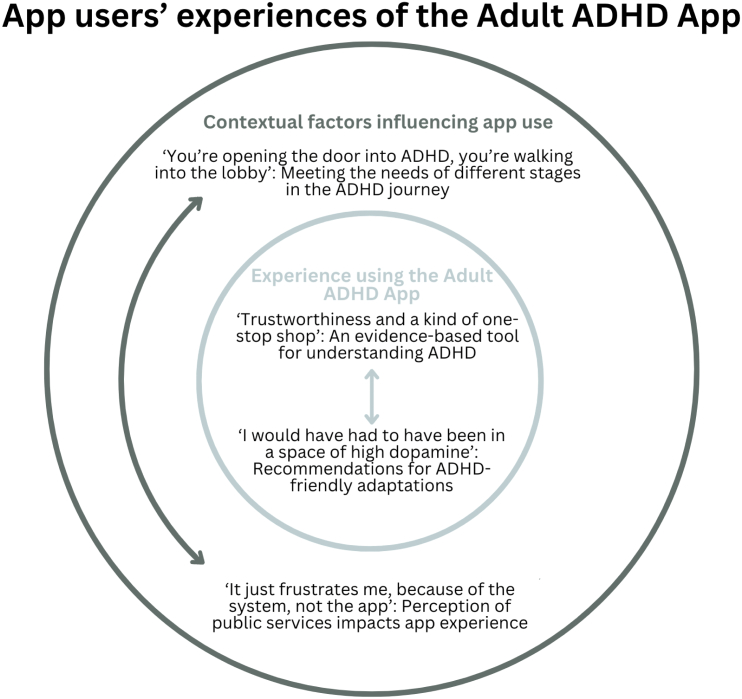
Visualisation of relationships between themes.

Four themes were developed using reflexive thematic analysis. The themes reflect participants' experiences when using the app and the contextual factors that influenced their expectations and needs from the app. Fig. 3 provides a visualisation of how the themes are related to one another, while Table 1 showcases the codes attached to each theme.

**Table 1:** Overview of codes associated with themes.

Theme	Codes
1. An evidence-based tool for understanding adult ADHD.	Positive response to information; trustworthiness; lived experience; therapeutic effects; ideas for other content
2. Recommendations for ADHD-friendly adaptations.	Navigation; density; overwhelm; app visuals; length of content; videos; gamification/reward system; engaging features; notifications; planner; mindfulness breaks
3. Meeting the needs of different stages in the ADHD journey.	A tool for beginners; motivation for use; the app as a resource to educate others
4. Perception of public services impacts app experience.	Perception of healthcare services; personal engagement with HSE; information on HSE; Irish specific; ADHD Ireland; credibility of developers

#### 3.2.1 ‘Trustworthiness and a kind of one-stop shop’: An evidence-based tool for understanding adult ADHD

The app appeared to meet its objective of providing evidenced-based psychoeducation to adults with ADHD with the “*scientific approach*” (Participant 2, 47, man) supporting the app's credibility:

“There seems to be an awful lot of unhelpful, I suppose, information out there, and information of dubious quality. So the app at a simplest, there is an element of kind of trustworthiness and a kind of a one-stop shop” (Participant 1, 45, man).

The app provided participants with information on a range of different topics: “*There's lots of stuff in there depending on the frame of mind you're in*” (Participant 3, 65, woman). Participants found the information on ADHD traits, self-help, and living with ADHD most valuable, in comparison to content about getting a diagnosis, interventions and negotiating the public health system. This may reflect that most participants were previously diagnosed, while information on ADHD traits and living with ADHD would always be relevant.

Some participants noted the positive effects of the psychoeducational content. For one participant, discovering the app helped him foster self-compassion and understanding of his ADHD traits: “*So huge relief that I wasn't mad, or loser or some sort of just weirdo… It's been really, really positive*.” (Participant 2, 47, man). Other participants resonated with tools recommended in self-help techniques, such as mindfulness practices.

While the psychoeducation was a valuable resource, some participants identified the lack of content on lived experience of having ADHD as a limitation. One participant suggested a community forum (Participant 4, 39, man), while others recommended video testimonials or case studies that highlight the positive elements of ADHD:

“Getting ordinary people, not doctors and not clinicians, and not psychiatrist and all that kind of stuff. No offence to you guys, but like, yeah, but just ordinary people” (Participant 2, 47, man).

#### 3.2.2 ‘I would have had to have been in a space of high dopamine’: Recommendations for ADHD-friendly adaptations

Participants recommended some changes to increase the app's appropriateness for adults with ADHD. Participant 11 (36, woman) described that to engage with the app as it is, she “*would have had to have been in the space of having high levels of dopamine, been in a really good mood, had a lot of energy and time*.” The primary limitation was navigating the app. Participants outlined two key issues with the app's layout – not knowing where to start and becoming overwhelmed at the number of pages they could read. This could lead to a sense of becoming lost in the app.

Some participants suggested alternative navigation structures, such as organising the app in terms of “*question, solution, question, solution*” (Participant 4, 51, man) to make finding information easier. There were also some suggestions to gamify content to reduce the feeling of needing to connect with all the information at once:

“But I think if it had been sort of like and then for less lesson plans or little stages that a little nugget opened each step at a time, so lesson one, then into lesson two. I think I would have been much more likely to go back on, because I would say ‘right, I've done this section, I need to do the next one’” (Participant 10, 30, woman).

Adding a gamified element could give the ‘dopamine hit’ multiple participants felt the app was lacking, which might encourage them to use the app more. Rewards, badges or streaks for engaging with mindfulness practices or reading psychoeducation content were also noted as ways to improve engagement. These suggestions related to participants' ideas to incorporate a notification system to motivate continued use: “*Reminders like that's like a massive thing like even for me – and like reminders to do like silly things like have lunch or drink water, or what are my plans…*” (Participant 14, 32, woman).

Participants preferred pages with colourful graphics and suggested having more graphics throughout, as well as increasing the visual accessibility of the app.

#### 3.2.3 ‘You're opening the door into ADHD, you're walking into the lobby’: Meeting the needs of different stages in the ADHD journey

Most participants felt the app was most useful for adults who were questioning if they have ADHD or who have been recently diagnosed: “*If I put myself into my shoes like when I had been diagnosed like this would have made a big difference*” (Participant 11, 36, woman). The app offered an introduction to ADHD which helped to foster their interest and a starting point for learning more:

“You know, if you didn't have the app your journey into discovery about ADHD would be very kind of chaotic ... It's like, you know, you're opening the door into ADHD, you're walking into the lobby” (Participant 2, 47, man).

In comparison to those ‘new’ to their ADHD, participants with more experience of ADHD appeared to have a different motivation for using the app. Instead of wanting to gain understanding about ADHD, they seemed to want an app that would help them in moments of challenge: “So you'd normally go into it – well for me anyway – I'd go into it when I really like I'm really struggling with something” (Participant 4, 39, man). The mindfulness breaks were often mentioned as a support in moments of difficulty, with participants finding value in brief practices targeted for ADHD.

Some participants recommended further content on how family and friends can support a loved one who has ADHD, highlighting that app users would like to be able to engage the people in their lives in discussions about their ADHD with the support of psychoeducational material. These findings suggest adults with ADHD engage with the app in different ways depending on where they are in their ADHD journeys.

#### 3.2.4 ‘It just frustrates me, because of the system, not the app’: Perception of public services impacts app experience

Participants often related the app to their own experiences and perceptions of the public health services in Ireland. For some participants, they were “*pleasantly surprised*” (Participant 1, 45, man) at the quality of the app as a product of the HSE, potentially reflecting low expectations of the app ahead of using it.

Other participants described finding the app, particularly information about ADHD services available in Ireland, as triggering or an unrealistic account of how to access support. It was important to some participants that the content about services was not “getting people's hopes up” when they perceived it as inaccessible (Participant 4, 39, man):

“like because the way the system is here and the waitlists are so long and so on it just frustrates me, because of the system, not because of the app. So some of this, like some of this, I find actually more triggering because– but because of the system here” (Participant 12, 43, woman).

There seemed to be a divide in how participants viewed the role of the HSE in developing the app. Some app users also found the emphasis in the app on the HSE as off-putting: “*Then there's a flash of HSE sign which gives me shivers. I have a negative bias towards it, for whatever reason*” (Participant 9, 56, woman). In comparison, others found it added credibility to the app. This view of the app as being developed by a reliable source was further supported by ADHD Ireland's role in collaborating to create the app. In contrast to the public health system, participants described having positive experiences with ADHD Ireland, and that this fostered interest and trust in the app. These findings highlight the value of working with community-based non-profit associations to develop and deliver digital mental health resources:

“[ADHD Ireland's] users are using it. I've had really good experiences with their community, so I'll engage with it as well” (Participant 5, 26, woman).

## 4 Discussion

The Adult ADHD App is a psychoeducational resource on adult ADHD and related services in Ireland and is available to download in the App Store and Google Play Marketplace. The present study sought to present patterns of real-world use of the app and offer insight into app users' experiences via a qualitative investigation with semi-structured interviews. The audit of app analytical data showed the app has been downloaded over 18,200 times over a period of one year, with 12,000 users and 6400 users who returned to the app. The average number of engaged sessions was 2.4 per user. Reflexive thematic analysis generated four themes that suggested participants found the psychoeducation valuable, the need for adjustments to improve usability, the benefits for adults newly learning about ADHD and the impact of attitudes and experiences of the health system in Ireland on app use.

The audit of the Adult ADHD App analytics demonstrated that the app had been downloaded over 18.2 k times, with 12 k active users and 6.4 k people returning to the app. This retention rate of approximately 50 % appears to reflect other digital mental health apps. For example, of the 158,930 people who downloaded MoodTools, an app for depression, 51.14 % returned to the app after initial download (Su and Anderson, 2022). Users of the Adult ADHD App engaged with the app for an average of 2.4 sessions, for approximately nine minutes in total. This is similar to MoodTools, where typical use was three sessions for a total of 12 min. Likewise, an app for people with bipolar disorder observed that only 28 % of their originally active users continued to use the app after 6-months (García-Estela et al., 2022). A systematic review of self-help digital interventions for mood found that completion or sustained use rates vary from 0.5 % to 29 % (Fleming et al., 2018). The app return rate may indicate good intervention coherence and perceived effectiveness. However, there may be modifications that can improve user experience, as highlighted by the qualitative investigation. While the 14 participants may not represent all of the views of the 12 k users who engaged with the app, their experiences can provide insight into priorities for improvement.

‘An evidence-based tool for understanding adult ADHD’ highlights the importance of psychoeducation to adults with ADHD, as they found significant value in the diversity of content of the app and knowing the app adopted a scientific, evidence-based approach. The value of having evidence-based information is highly important given the rising interest in ADHD online and concerns about misinformation (Hartnett and Cummings, 2023). For example, 52 % of the top most popular videos about ADHD were classified as misleading (Yeung et al., 2022). A qualitative study on the perception of adults with ADHD on psychoeducation while waiting to access services found participants felt receiving psychoeducation would be empowering (Gore et al., 2023). This is evidenced by participants in the present study finding the app helped reduce feelings of isolation and offered self-help techniques like mindfulness.

Participants recommended changes to the app to improve usability within ‘Recommendations for ADHD-friendly adaptations’. The greatest challenge of the app was navigating through it, as participants sometimes felt they did not know where to start or could become overwhelmed with the amount of content. Kenter et al. similarly had difficulties with the usability of navigation and functionality in an online self-help intervention for adults with ADHD (Kenter et al., 2022). One suggestion from participants to address the navigation was to incorporate gamified elements by ‘unlocking’ content, which could be linked to the need for positive re-enforcement and reminders. It may be particularly helpful for adults with ADHD to have gamified elements within apps to encourage use and sense of accomplishment, as other adults with ADHD have recommended reward-systems for digital mental health tools (Seery et al., 2022).

The value of the app for adults who had recently discovered their ADHD was highlighted in ‘Meeting the needs of different stages in the ADHD journey’. Adults with ADHD can feel they do not know enough about ADHD and seek to obtain more information through pamphlets, books, and the internet (Aoki et al., 2020). The app was likely most supportive for adults new to their ADHD as they could engage with that therapeutic process of understanding their ADHD and connect with a breadth of evidence-based psychoeducation on ADHD. This is in line with the aims of the app as an introductory psychoeducational resource for adult ADHD. While this is how the app is advertised, most participants were further along in their ADHD journey and still sought out the app, although they were hoping for more advanced content and a tool to support them in moments of difficulty. In previous research, adults with ADHD have highlighted the importance of recognising the diversity within ADHD and adapting interventions appropriately (Oscarsson et al., 2022), suggesting digital health interventions may also need to target adults at different stages of familiarity with their ADHD. Therefore, while the Adult ADHD App appears to meet its goals as an introduction to psychoeducation on ADHD, it is clear that ongoing supports are needed for adults with ADHD beyond the post-diagnostic period. For individuals who need more psychoeducation and therapeutic input beyond the app, we developed and evaluated the Understanding and Managing Adult ADHD Programme (UMAAP) (Seery et al., 2024). The qualitative evaluation of UMAAP found that participants experienced the psychoeducation in UMAAP as novel, with many new concepts, and the Acceptance and Commitment Therapy particularly valuable. It may be that the Adult ADHD App is an effective introduction for adults newly on their journeys, while UMAAP is a more beneficial intervention for individuals with more experience with their ADHD. Further research is needed to understand what level of psychoeducation would be most helpful for individuals several years post-diagnosis.

‘Perception of public services impacts app experience’ explored the complex dynamics of providing a digital health tool developed by a public health system. This has similarly been shown by other research evaluating the COVID-19 Contracting Tracing app developed by the National Health Service (NHS), the public health system in the United Kingdom, where participants with lower trust in the NHS had more negative views of the app (Dowthwaite et al., 2021). In the present study, participants' experiences of the public health system in Ireland seemed to influence their attitudes towards content on the app. Previous research has demonstrated that individuals with ADHD experience challenges in accessing assessments and services (Oscarsson et al., 2022; Long and Coats, 2022). Given the challenges adults with ADHD can have in accessing care and that personal experience of care can influence engagement, digital health tools developed by public health services should aim for sensitivity when providing information about supports available.

In summary, the real-use data and qualitative findings provide insight into acceptability in several ways. The theme ‘Perception of public services impacts app experience’ indicated that some participants were surprised at the quality of the app given its association with the HSE, which might suggest some difficulties with prospective acceptability and perceived effectiveness, although the number of downloads is suggestive of positive prospective acceptability. User retention could be suggestive of concurrent acceptability, although ‘Recommendations for ADHD-friendly adaptations’ highlighted the potential burden of the app and difficulties with its suitability, potentially reducing the self-efficacy of users. The themes ‘An evidence-based tool for understanding adult ADHD’ and ‘Meeting the needs of different stages in the ADHD journey’ support the perceived effectiveness of the app, as participants described learning from the varied trustworthy psychoeducation and recommended the app as a tool for adults new to their ADHD.

### 4.1 Limitations and future research

The study's findings should be considered in relation to its limitations. We were limited in what app analytical data we could access based on the data the app collected. For example, we could not investigate the demographic factors associated with attrition in use, which provided valuable context for use of an app for people experiencing bipolar disorder (García-Estela et al., 2022). It would be beneficial to explore the association between demographic factors and use of digital health tools in adults with ADHD. Additionally, only average and rounded figures were available for the data, which limited insight into app use. Approximately 12,000 people actively used the app, with fourteen individuals interviewed. There are no specific requirements for the number of participants required for qualitative research, and sample sizes are instead impacted by the discipline, journal norms, time and financial constraints, and an acceptable level deemed by the researchers (Braun and Clarke, 2021). While a sample size of fourteen participants is not atypical in the context of qualitative research, in the context of the many people who downloaded the app, it is small and the views expressed by the present study's participants do not necessarily reflect the 12,000 who have downloaded the app, or the 51 % who returned to the app. An open-ended survey may have increased participation in the qualitative component of the study, allowing for further views on experiences of the app, although likely would have still been a small sample compared to the number of app downloads.

Additionally, details on participants' experiences of medical and psychological interventions as well as previous use of mobile apps for ADHD or mental health difficulties were not collected. Previous research has identified that consistent use of medication during behavioural therapy is associated with larger improvements in adolescents with ADHD (Sibley et al., 2022). As such, it would be helpful for future studies to collect information on participants' medication use, experiences of psychological interventions, and previous engagement with apps to contextualise their responses. For instance, if individuals currently engaged with medical or psychological interventions had different views on the HSE as a contributor to the Adult ADHD App.

Furthermore, a challenge in evaluating passive psychoeducation, such as the Adult ADHD App, is that the researchers cannot be sure how much the participants read through or engaged with the content (Brooks et al., 2021). Qualitative participants were not asked specific questions about their average usage duration and how frequently they used the app. Like Arnold et al. (Arnold et al., 2020), we asked participants to tell us about a time that they used the app (with probes of time, place, device used). However, no participants were able to give specific details about use, potentially reflecting the working memory differences linked to ADHD (Alderson et al., 2013). It might have been challenging for participants to estimate their usage. However, without this information, we cannot be sure if the participants' depth of engagement is comparable to one another or the average user engagement indicated by the audit of app analytical data. Future studies exploring qualitative experiences of an app should include questions for participants to estimate their use, with awareness of executive functioning differences that might impact on answers when exploring the experiences of neurodivergent people. Additionally, a self-report scale of engagement might be beneficial in contextualising qualitative feedback. We recommend consulting Bijkerk and colleagues (Bijkerk et al., 2023), who found multiple self-report scales to assess engagement in their systematic review, such as the Digital Behaviour Change Interventions (DBCI) Engagement Scale (Perski et al., 2019), which measures levels of interest, intrigue, focus, and pleasure when engaging with an intervention.

Future research could evaluate the effectiveness of psychoeducation on the app, potentially targeting those new to their ADHD as they appeared to benefit the most from the app.

Finally, the study did not adopt a ‘think-aloud’ method, in which participants share their thoughts aloud while they engage with written content of an intervention (Eccles and Arsal, 2017; Yardley et al., 2021). However, multiple participants opened the app during the interview to prompt themselves anyway. Future qualitative evaluations of digital health tools for adults with ADHD could consider incorporating the ‘think-aloud’ method, as it could support adults with ADHD's memory-related differences (Alderson et al., 2013).

## 5 Conclusion

The Adult ADHD App was launched in November 2022 and aims to provide psychoeducation about adult ADHD. Our findings showed the app has been downloaded over 18.2 k times, with 12 k active users. Approximately half of users return to the app, which reflects user retention of other digital mental health apps. The Adult ADHD App appears to provide helpful psychoeducation and is particularly beneficial for adults who are newly diagnosed. Findings highlighted areas for improvement of usability. Additionally, experiences of the public health system impacted attitudes towards the app. As such, findings should be used to improve the app in terms of content, such as minimising information the public health system, and to make the features more accessible for ADHD. Overall, the present study demonstrates that while changes are needed to improve suitability, the app appears to meet its aim of providing credible psychoeducation for individuals with ADHD, especially those recently self-identified or diagnosed.

## Declaration of competing interest

The authors declare that they have no known competing financial interests or personal relationships that could have appeared to influence the work reported in this paper.

## Data Availability

The qualitative data is available upon reasonable request. The app analytical data is not available for further use.

## Appendix A Supplementary data

Supplementary material Image 1
