# Supplementary material for: “A one-stop shop”: Real-world use and app-users' experiences of a psychoeducational smartphone app for adults with ADHD
Source: Internet Interv. 2025 Feb 4;39:100807. doi: 10.1016/j.invent.2025.100807 (PMC11847728; doi:10.1016/j.invent.2025.100807)
Supplement: Supplementary file 1 — Supplementary material [file mmc1.docx]

**Interview Schedule**

The interview schedule is provided for reference. It was adapted from Arnold C, Williams A, Thomas N. Engaging With a Web-Based Psychosocial Intervention for Psychosis: Qualitative Study of User Experiences. JMIR Mental Health 2020;7:e16730. https://doi.org/10.2196/16730.

| **Prior to interview** | Ensure that the participant has read the information sheet and consented to participate. |
| --- | --- |

| **Introduction**  **Consent**  **Explain purpose of the study and take any questions**  **Demographic Questions** | Introduce self, welcome, and thank participant for attending the interview. Ensure participant is comfortable.  Re-confirm informed consent is still valid and participant still wishes to take part.  Thanks again for meeting with me today. The interview will focus on your general experience of using the Adult ADHD App, factors influencing App use, App features, the self-help techniques on the app and if or how you engage with the content outside of being on the app. The interview will take around 20-30 minutes to complete, or until you’re finished talking. With your permission, the interview will be recorded and then will be typed. When it is typed, the recording will be deleted. Quotes that you provide during the interview may be included in publications and the main researcher’s PhD dissertation. Do you have any questions at all?  Ask for demographic data (age, gender, have you used the app – if so, how often, nature of app use (do you have ADHD yourself, does someone you know have ADHD?, are you a clinician?) |
| --- | --- |

| **Focus 1: General experience of using the HSE adult ADHD App** | How did you find using the App?  Can you tell me about a time that:  You used the App  Probe: time, place, device used, alone/with someone  Something prevented you from using the App |
| --- | --- |

| **Focus 2: Factors influencing use of the resources** | What made you start using the Adult ADHD App?  How did you hear about it?  What, if anything, helped you continue to use the App after you started?  What, if anything, prevented you from using the App or made it hard to use?  What, if anything, made the App easy to continue using?  What, if anything, made you stop using the App? |
| --- | --- |

| **Focus 3: App pages** | Were there any pages of the App you came back to?  Did those pages have any influence on your use of the App? Or experience of using the App?  Were there any pages you preferred not to use?  Were there any pages you felt should be added? |
| --- | --- |

| **Focus 4: Self-help techniques** | On some pages, there are some self-help tips under the subheading, ‘what can I do about it?’ – what did you think of these sections?  What, if anything, was helpful about these tips?  What, if anything, was unhelpful about these tips?  Were there any tips you started to use yourself?*  *only ask if participant is an ADHDer (diagnosed or self-identified) |
| --- | --- |

| **Focus 5: Engagement with content outside website use** | Did you have any specific goals related to using the App?  Have you noticed any changes in your life since you started using the App?  If so, what do you think may have influenced those changes? |
| --- | --- |

| **Interview Closedown** | Is there anything else that you would like to tell me that we haven’t discussed, but you think might be relevant when evaluating the Adult ADHD App?  How have you found this interview today? Is there any way this interview could be improved for future participants?  Okay I’ll now switch off the recording. |
| --- | --- |

| **End of interview** | Thank the participant for taking part.  Explain what will happen with the information provided – when all the interviews are completed, they will be analysed to see if there are any patterns or contradictions in the data. It’ll then be written up for publication and as part of the main researcher’s PhD.  Ask the participant whether they have any questions. |
| --- | --- |
